# Supplementary material for: Is There a Foreign Accent Effect on Moral Judgment?
Source: Brain Sci. 2021 Dec 10;11(12):1631. doi: 10.3390/brainsci11121631 (PMC8699611; doi:10.3390/brainsci11121631)
Supplement: Supplementary file 1 [file brainsci-11-01631-s001.zip › brainsci-1452966-supplementary.pdf]

Supplementary Materials

Table S1. Dilemmas in English with their Spanish and Dutch translations

	Footbridge dilemma	Trolley dilemma
**English**	A train is going down a track very fast towards five people. The train has a problem and cannot be stopped, unless a heavy weight is dropped on the track. You are on a bridge and there is a very fat man next to you - your only way to stop the train is to push him onto the track, killing him to save five people.<br>Would you push him?	A train is going down a track very fast towards five people. The train has a problem and cannot be stopped. Five people will die if you stay on this track. There is another track that you can use to divert the train. At the end of this track there is one man that will die if you change the track.<br>Would you change the track?
**Spanish**	Un tren baja muy rápido acercándose a cinco personas. El tren tiene un problema y no se puede parar, a no ser que un objeto pesado sea lanzado a la vía. Estas en un puente y hay un hombre muy gordo cerca de ti- la única manera que puedes parar el tren es empujándole a la vía, matándole a él para salvar cinco personas.<br>¿Lo empujarías?	Un tren baja muy rápido acercándose a cinco personas. El tren tiene un problema y no se puede parar. Cinco personas morirán si te quedas en esta vía. Hay otra vía que puedes utilizar para desviar el tren. Al final de esta vía hay un hombre que morirá si cambias de vía.<br>¿Cambiarías de vía?
**Dutch**	Een trein rijdt heel snel op vijf mensen af. De trein heeft een probleem en kan alleen maar stoppen als er een zwaar object op het spoor wordt gegooid. Jij staat op een brug en er staat een hele dikke man naast je. De enige manier om de trein te stoppen is door hem op	Een trein rijdt heel snel op vijf mensen af. De trein heeft een probleem en kan niet remmen. Vijf mensen zullen doodgaan als je op dit spoor blijft. Er is een ander spoor dat je kunt gebruiken om de trein om te leiden. Op het einde van dit spoor staat één man en hij zal doodgaan als je de trein van spoor laat wisselen.

	het spoor te duwen. Hierdoor sterft hij, en red je de vijf mensen.<br>Zou je hem duwen?	Zou je het spoor wisselen?
